# Pyrolysis of wastewater sludge and composted organic fines from municipal solid waste: laboratory reactor characterisation and product distribution

**DOI:** 10.1007/s11356-018-1463-y

**Published:** 2018-02-26

**Authors:** David A. Agar, Marzena Kwapinska, James J. Leahy

**Affiliations:** 1 Department of Forest Biomaterials and Technology, Swedish University of Agricultural Sciences, SE-90183 Umeå, Sweden; 2 Department of Chemical Sciences, University of Limerick, Castletroy, V94 T9PX, Limerick, Ireland

**Keywords:** Waste valorisation, Pyrolysis, Sewage sludge, Municipal solid waste, Syngas, Biochar

## Abstract

Sludge from municipal wastewater treatment plants and organic fines from mechanical sorting of municipal solid waste (MSW) are two common widespread waste streams that are becoming increasingly difficult to utilise. Changing perceptions of risk in food production has limited the appeal of sludge use on agricultural land, and outlets via landfilling are diminishing rapidly. These factors have led to interest in thermal conversion technologies whose aim is to recover energy and nutrients from waste while reducing health and environmental risks associated with material re-use. Pyrolysis yields three output products: solid char, liquid oils and gas. Their relative distribution depends on process parameters which can be somewhat optimised depending on the end use of product. The potential of pyrolysis for the conversion of wastewater sludge (SS) and organic fines of MSW (OF) to a combustion gas and a carbon-rich char has been investigated. Pyrolysis of SS and OF was done using a laboratory fixed-bed reactor. Herein, the physical characterisation of the reactor is described, and results on pyrolysis yields are presented. Feedstock and chars have been characterised using standard laboratory methods, and the composition of pyrolysis gases was analysed using micro gas chromatography. Product distribution (char/liquid/gas) from the pyrolysis of sewage sludge and composted MSW fines at 700°C for 10 min were 45/26/29 and 53/14/33%, respectively. The combustible fractions of pyrolysis gases range from 36 to 54% for SS feedstock and 62 to 72% from OF. The corresponding lower heating value range of sampled gases were 11.8–19.1 and 18.2–21.0 MJ m^−3^, respectively.

**Electronic supplementary material:**

The online version of this article (10.1007/s11356-018-1463-y) contains supplementary material, which is available to authorized users.

## Introduction

There is growing interest in the use of thermal conversion technologies for waste management (Syed-Hassan et al. 2017; Kumar and Samadder 2017). These generally aim to valorise waste streams while reducing risks associated with re-use of waste materials. Pyrolysis, the thermal conversion of a substance, is of interest in waste management because it can reduce health and environmental risks from problematic wastes (Lindberg et al. 2015; Trinh et al. 2013) while providing an avenue for the recovery of energy and nutrients (Buah et al. 2007; Song et al. 2014).

Two common and widespread waste streams are sludge from municipal wastewater treatment plants (SS) and the organic fine (OF) component from the mechanical sorting of municipal solid waste (MSW). Wastewater sludge is the organic by-product of municipal wastewater treatment. It consists of the solids, which are removed from wastewater during the treatment process. Treatment methods can be mechanical, biological or chemical (Haller 1995). Sludge from wastewater treatment plants is commonly applied to agricultural land as a fertiliser. The re-use of sludge is the most encouraged outlet, according to current EU waste policy objectives which also permit optional methods that provide the best overall environmental outcome.

Organic fines of MSW are an extremely heterogeneous material containing food waste, plastics, metals, paper and glass (Buah et al. 2007). After the screening of MSW, the fine material is routinely stabilised through controlled aerobic composting after which it is used as a cover material at landfill sites (RPS 2014).

The utilisation of these two waste streams is undergoing changes. Firstly, societal perceptions of risk and quality assurance schemes in food production have lessened the appeal of spreading treated sewage sludge on agricultural land. Secondly, in the Republic of Ireland and elsewhere, landfill sites are closing down. Therefore, the outlets for sewage sludge and organic fines are rapidly diminishing, and new solutions are sought for their safe handling and utilisation (Kim and Parker 2008).

### Pyrolysis

Pyrolysis decomposes organic materials into other products under inert atmosphere. Wood charcoal, which is produced from the pyrolysis of wood, is a familiar example. Char, however, is only one of the products of pyrolysis. The process also yields liquids (oils and tars) and gases (syngas). The distribution of pyrolysis products depends heavily on several process parameters whose influence follows a general trend for all organic feedstock (Wang et al. 2011). Harmful emissions from waste pyrolysis (Han et al. 2017) and undesirable product characteristics (Leng et al. 2015), however, are also parameter-dependent (Yu et al. 2016; Yuan et al. 2011; Zhang et al. 2017). Therefore, process parameters need to be optimised for a particular application to achieve the best overall benefits (Buah et al. 2007).

Temperature, residence time and heating rate are the main process parameters, but particle size of the feedstock and residence time of vapour-phase products are also important as these influence the contact between chars and gases, the extent of which affects char formation and the decomposition (cracking) of long-chain hydrocarbon gases (Mok and Antal Jr 1983a, b). Long vapour-phase residence times encourage char-forming reactions. It follows that process flow conditions, for example batch versus continuous processes and reactor configurations themselves, strongly influence char-vapour interactions.

The temperature used in pyrolysis can range from 220 °C, as in the partial pyrolysis of wood (Doat 1985), up to 900 °C (Chen et al. 2014; Lombardi et al. 2015). Feedstock residence time varies from just a few seconds up to several hours. Generally, high temperature and a long residence time favour gas and char production while minimising the production of oils (Basu 2010). The liquid yield is maximised in a temperature range of 450–550 °C (Syed-Hassan et al. 2017). Above this temperature, the volatile content of the feedstock undergoes further decomposition resulting in more gas production.

The rate of feedstock heating also strongly affects product distribution. Pyrolysis is classified according to its heating rate, spanning a vast range from slow to fast, from a few degrees per minute up to 650 °C min^−1^ (Chen et al. 2014). High heating rates require effective heat transfer within a reactor. Fast pyrolysis uses high heating rates to produce more vapour-phase products and decrease char yields (Bridgwater 2012). Liquid biofuel production utilises rapid heating and very short residence time of vapour-phase products to maximise production of oils and tars which can then be refined. Slow pyrolysis, on the other hand, is used to maximise char production, allowing pyrolysis gases to stay in contact with produced chars. Identifying the appropriate heating rate for a pyrolysis process depends both on the feedstock characteristics and on the end use application (Al Arni 2017).

The pyrolysis behaviour of sewage sludge is a topic of interest in recent years with most investigations focusing on maximising oil yield (Fonts et al. 2012; Gao et al. 2014). A less common topic of study is the minimisation of oil production which can be aided through the use of an effective catalyst (Yu et al. 2017) and also through non-conventional heating of some feedstock types (Domínguez et al. 2007). Catalysts can reduce the need of a high operating temperature in a pyrolysis reactor which is normally required for greater gas production (Jaramillo-Arango et al. 2016). Moreover, char is a desirable product from the perspective of nutrient recycling. Minimising liquid yield may be desirable for a decentralised pyrolysis process aiming to utilise syngas on-site through direct combustion and simultaneously producing biochar for subsequent uses.

### Purpose

The purpose of the present study was to investigate the potential of pyrolysis as a conversion technology for the distributed treatment of waste streams. In this context, pyrolysis was viewed as an alternative to incineration. Laboratory-scale pyrolysis experience and reactor characterisation are to be used to optimise a pilot-scale process currently under development with technological partners. Initial investigations focused on the syngas and char potential of waste by determination of yield distribution from pyrolysis. Additionally, feedstock, char and gas fractions were to be characterised. This was a starting point for enhancing the process for energy and/or nutrient recovery and transposing results to a commercial process. This work formed part of a 32-month national project, launched in 2016, on the feasibility of pyrolysis for waste management.

## Materials and methods

### Feedstock materials

**Fig. 1:**
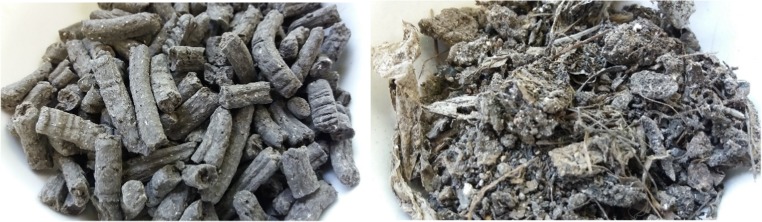
Photographs of feedstock materials. Sewage sludge (SS) pellets (left) and composted organic fines (OF) from municipal solid waste (right)

Two feedstock types were used in laboratory investigations: sludge from a municipal wastewater treatment plant (SS) and composted organic fines from MSW (OF). SS samples were pre-dried and in the form of pellets, supplied by Northumbrian Water, Co. Cork, Ireland. They had a moisture content of 9.8% (wb). The average pellet size was approximately 10 mm in length and 4 mm in diameter (Fig. 1). Pyrolysis was also carried out on SS samples ground to a 1-mm size (20 g 700 °C) to determine the influence of particle size on yields.

OF samples were supplied by Greenstar, Co. Cork, Ireland. They had a maximum particle size of about 10 mm and had a moisture content of 8.5% (wb) after air-drying. The as-received moisture content varied between 17 and 25% (wb). Glass, which can make up to 20% mass of the sampled material, and metal components were removed from the feedstock.

### Experimental apparatus

The pyrolysis tube reactor (supplementary figure) consists of a cylinder (45/50 mm, ID/OD) of quartz (H. Baumbach & Co. Ltd., UK) whose exterior was evenly wrapping with Samox® heavily insulated heating tape whose maximum power output was 940 W (Omegalux, USA). Two layers of woven high-temperature insulation, having a combined thickness of approximately 10 mm, were wrapped on the outside of the heating tape and secured with aluminium foil tape at either end. A model MC227 electrothermal power regulator (Cole-Parmer, UK) supplied the heating tape with AC electricity. The total length of the reactor cylinder is 600 mm and that of the heated section is approximately 350 mm. The reactor was held in place using clamps attached to two or more ring stands and was inclined from the horizontal by approximately 10°.

**Fig. 2:**
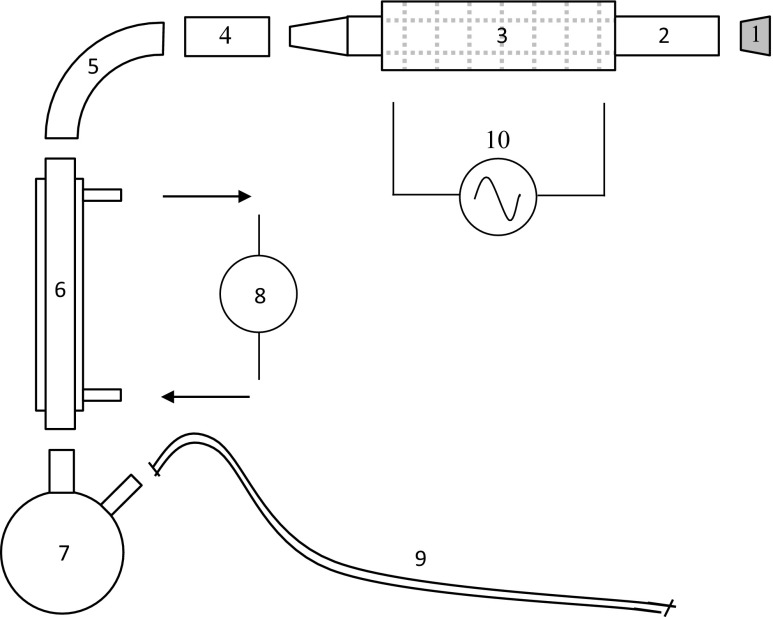
Exploded view of laboratory pyrolysis apparatus (not to scale). Rubber stopper (1), quartz reactor tube (2), heated and insulated section of reactor tube (3), reducer (4), 90° bend (5), condenser (6), twin-neck round-bottom 500-ml flask (7), refrigerated liquid controller (8), exit tube for gas sampling (9), power regulator (AC) (10)

Borosilicate (Pyrex®) jointed glassware, manufactured by Quickfit®, formed the remainder of the apparatus (Fig. 2). One end of the reactor tube was open and sealable with a removable rubber stopper. The other end of the cylinder tapered to a ground glass fitting to match a reducer fitting (XA43). A 90° bend then followed, leading to the condenser section (C1/13/SC) whose outer jacket was cooled via circulation of a refrigerated liquid maintained at a temperature of 268 K. The condenser section was inclined from the horizontal by approximately 45°. A twin-neck round-bottom 500-ml flask was connected below the condenser to hold pyrolysis liquids. A 10-mm (OD) rubber tube, approximately 50 cm in length, was connected to the other neck of the flask above the liquid level. The other end of this tube was open to atmosphere and fitted with a plastic connector, suitable for attaching gas sampling bags.

Temperature was measured using a sheathed K-type thermocouple of 1000-mm length which was inserted into the reactor via a feed-through hole in the rubber stopper. The hold was sealed on the outer side of the stopper using a small amount of Blu Tack®. Temperature was read within 1 °C using a digital handheld display (VWR).

Feedstock samples were loaded into a purpose-made cylindrical steel-mesh basket having a length of about 250 mm and slightly smaller diameter than that of the inside of the reactor. The mesh size was 0.25 mm.

### Experimental procedure

The relative distribution of pyrolysis projects was determined by the principle of conservation of mass—the combined mass of pyrolysis products is equal to that of the initial sample feedstock. The primary assumption is that no liquids exit the apparatus in the vapour phase; all condensable products are present.

Before each pyrolysis run, the reactor, the connected glassware, the empty sample basket and the rubber stopper were weighed to within ± 0.01 g. A sample of feedstock was poured into the basket, and the basket was weighed again. Fine particles (< 380 μm) were screened from the sample. Pyrolysis runs were carried out using 20 and 50 g samples at 600 and 700 °C.

The reactor and glassware were assembled in a fume hood, and the heating tape leads were connected to the power regulator and switched on. Ground glass connections were sealed with a few wraps of Teflon® tape. The condenser coolant tubes were connected and circulation of the refrigerated liquid commenced. After approximately 50 min, the reactor reached a steady state and was ready for use.

The thermocouple was inserted through rubber stopper to a suitable length so that its tip was located in the middle of the heated section of the reactor tube. The sample basket and thermocouple together were inserted into the open end of the reactor in one smooth motion ending with the stopper being in place. The timer was started and the fume hood closed. After 10 min, the heating was switched off. After 30 min, the cooling was switched off. The reactor tube cooled to room temperature after about 2 h. The sample basket and contained char were removed from the reactor and weighed. The reactor, the glassware (containing condensed and liquid pyrolysis products), the empty basket and the rubber stopper were weighed again.

The initial sample mass was calculated as the difference between the empty and loaded basket before pyrolysis. The mass of char was calculated as the difference between the empty and loaded basket after pyrolysis. The mass of pyrolysis liquid was calculated as the difference in mass of the apparatus (reactor and all components) before and after pyrolysis. The mass fraction of gas was calculated as the difference between 100% and the char and liquid mass. The mass balance was calculated on a dry mass basis—water originating in the sample was subtracted. Therefore, the liquid fraction of pyrolysis products consists of oil, tar and water formed only through decomposition reactions.

### Physical characterisation of the reactor

There are a number of physical quantities used to characterise a pyrolysis reactor. These describe heat transfer and operational parameters of the pyrolysis process.

The heating rate *χ* (K s^−1^) of a pyrolysis reactor is equal to the effective heating power *P* (J s^−1^) divided by the heat capacity *C* (J K^−1^) of the feedstock:

1 $$ \chi =\frac{P}{C} $$

*P* is the product of the heat flux *q* (W m^−2^) and heat transfer surface area *A* (m^2^) of the reactor wall (Eq. 2). The heat flux is the product of the temperature difference Δ*T* (K) between the wall of the reactor and the feedstock and the heat transfer coefficient *α* (W m^−2^ K^−1^) within the reactor (Eq. 3). *C* in Eq. 1 is the product of feedstock mass *m* (kg) and its specific heat capacity *C*_*p*_ (J kg^−1^ K^−1^).

2 $$ P=q\times A $$

3 $$ q=\propto \times \Delta  T $$

The heat transfer coefficient is found by combining the above equations and solving for *α*, which yields Eq. 4.

4 $$ \alpha =\frac{\chi \times m\times {C}_p}{A\times \Delta T} $$

With the possible exception of *A*, the factors in Eq. 4 are strictly a function of time. The heating rate *χ* and Δ*T* are determined experimentally. *χ* is equivalent to the slope of the temperature versus time profile of the heated feedstock. By measuring the reactor wall temperature simultaneously, Δ*T* is determined.

The dimensionless *Biot* number *Bi* is determined within the reactor by the use of Eq. 5, in which *r*_*p*_ (m) is the feedstock particle diameter (assumed to be spherical) and *λ* (W m^−1^ K^−1^) is the thermal conductivity of the feedstock.

5 $$ Bi=\frac{\alpha \times {r}_p}{\lambda } $$

The Biot number is the ratio between the rate of heat convection (numerator) and conduction (denomination). A *Bi* value appreciably smaller than 1 indicates that heat transfer within the feedstock is rapid enough and resulting char is evenly cooked across the particle and that thermal control of the reactor is achieved.

### Temperature profile, residence time and assumptions

**Fig. 3:**
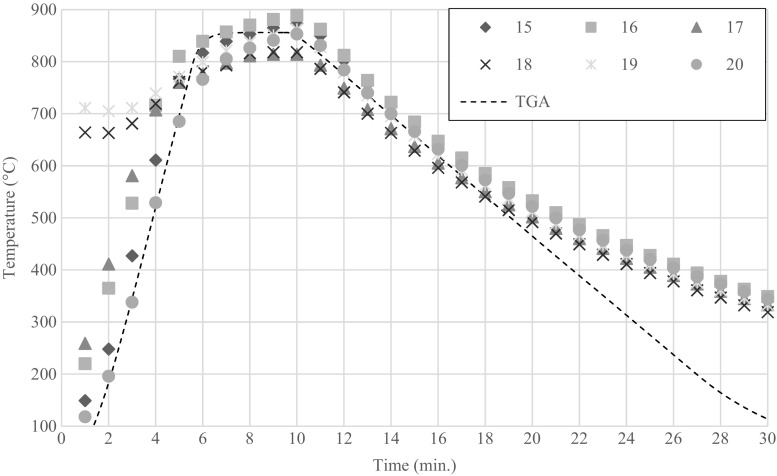
Measured temperature of SS feedstock (15, 16, 17, 20) and reactor wall (18, 19) during six different pyrolysis runs (20 g at 700 °C). The TGA simulated heating profile used for mass loss determination (dashed line)

SS feedstock was used to characterise the pyrolysis reactor because of its homogeneity in composition. The temperature profile within the reactor was measured as a function of distance from the internal wall of the quartz tube reactor. This varied from directly adjacent to the wall (outside the sample basket) to completely within the sample. To determine the variation in temperature between the reactor wall and the sample, an average temperature profile from several pyrolysis runs was calculated (Fig. 3). Temperature profiles using OF feedstock showed much greater variation between individual runs.

The two most extreme temperature profiles, that of the reactor wall and that within the bulk of the sample, were used to calculate Δ*T* and the resulting heat transfer coefficient. This value is interpreted as a minimum value for this process because *α* is inversely proportional to Δ*T* (Eq. 4). The corresponding Biot number was then calculated.

The residence time of a sample was determined through averaging the temperature data from several runs. For a 20 g sample, the residence time was 9.8 min at 700 °C and 6.2 min at 800 °C. A 50 g sample had a residence time 8.2 min at 700 °C and 4.1 min at 800 °C.

The change of mass of the feedstock during pyrolysis (Eq. 4) was estimated using a normalised mass loss curve from TGA analysis (PerkinElmer Pyris). The heating profile of the feedstock in the reactor was simulated in the TGA (Fig. 3) using a sample mass of 7.5 mg, initial heating rate of 150 K min^−1^ and nitrogen flow of 20 ml min^−1^. A value of 1950 J kg^−1^ K^−1^ was used for the heat capacity of dried sewage sludge (Kim and Parker 2008). For the thermal conductivity of the sludge, a value of 0.1 W m^−1^ K^−1^ was used.

### Characterisation of feedstock and pyrolysis char

Proximate analysis of feedstock and pyrolysis chars were carried out according to standard methods for heating value (EN 15400), ash content (EN 14775), moisture content (EN 15414) and volatile content (EN 15402). Ultimate analysis of the feedstock and char was performed using two samples for each material. The pyrolysis chars analysed were from SS (50 g, 700 °C) and OF (50 g, 700 °C). The sample size used in elemental analysis of chars was 60 mg.

### Pyrolysis gas analysis

Pyrolysis gas sampling was done using Tedlar® 0.5-l bags with a polypropylene valve (Restek, Ireland). The bags were filled via the exit tube of the reactor (Fig. 2). Gas samples were taken at regular intervals during the batch pyrolysis run with each sampling lasting from 15 to 30 s depending on the filling rate. Samples were analysed using a micro gas chromatograph (Agilent 3000).

The reactor tube contained air at the start of a run. To determine the extent of carbon oxidation in the sample due to oxygen present, the required stoichiometric volume (ideal gas) of air required for combustion (*C* + *O*_2_ → *CO*_2_) of the feedstock carbon was calculated. At 600 °C, the required volume of air was 152 l while the volume of the reactor tube is 1 l. Therefore, an inert atmosphere can be assumed.

The lower heating value *LHV* (MJ m^−3^) of the pyrolysis gas was calculated using the volumetric fraction *f*_*x*_ (dimensionless) of the combustible gas component *x* and the lower heating value of that component *LHV*_*x*_ (MJ m^−3^) as in Eq. 6 (Basu 2010).

6 $$ LHV=\sum {f}_x\times {LHV}_x $$

$$ LHV={f}_{CH4}\times 35.883+{f}_{CO}\times 12.633+{f}_{H2}\times 10.783+{f}_{C2H4}\times 59.457+{f}_{C2H6}\times 63.79 $$

## Results and discussion

### Heat transfer within the reactor

**Fig. 4:**
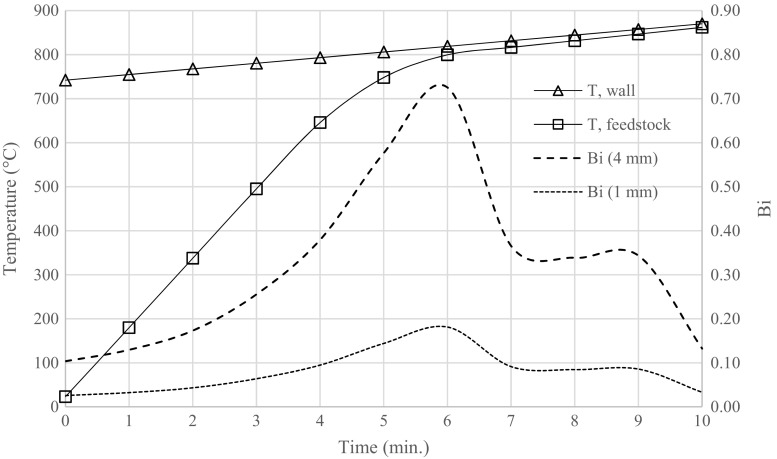
Modelled temperature profile of reactor wall and feedstock with calculated Biot number for 1 and 4-mm particle diameters

The temperature of the reactor wall and feedstock, whose difference represents Δ*T*, is depicted in Fig. 4 along with the calculated Biot number for 1 and 4-mm particle sizes. The heat transfer coefficient *α* ranged from 2.6 to 18.1 W m^−2^ K^−1^ for SS feedstock. The heating rate ranged from 160 to 5 K min^−1^. The undulating appearance of the Bi curve in Fig. 4 is a result of the difficulty of evaluating the heating rate *χ* (*dT*/*dt*) from the temperature curve. The important feature of the figure is that *Bi* has a value well below unity over the run, even for feedstock having a 4-mm particle diameter (SS). For 1-mm particle sizes, *Bi* decreases accordingly (Eq. 5). This indicates that heat transfer within the sample is rapid enough for sufficient thermal control during pyrolysis.

### Mass balance

**Fig. 5:**
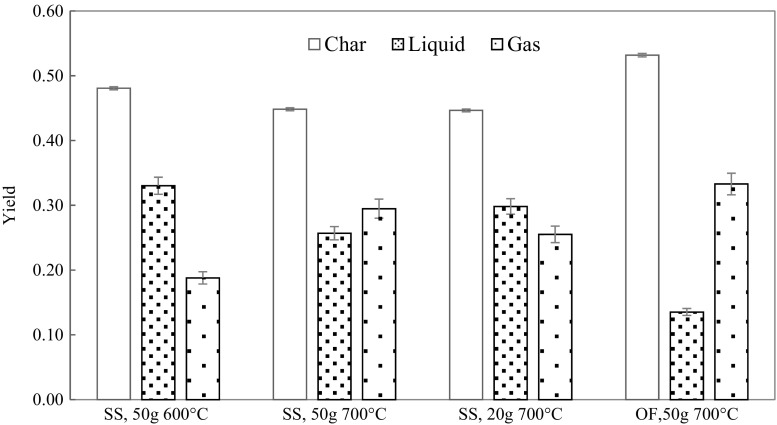
Mass balance of laboratory-scale pyrolysis with error bars

The distribution of pyrolysis products for four series of runs is shown in Fig. 5. Each series was an average of five separate runs whose standard deviations are given in Table 1. Focusing first on the differences between the two feedstock, the char yield for SS ranged from 45 to 48%, while that of OF was 52%. The liquid yield of SS runs had an average of 25–32% while that of OF was 14%. Correspondingly, the gas yield from OF was the highest observed being 33% while gas fractions from SS had a range of 19 to 29%.

**Table 1:** Mass yields of pyrolysis products including standard deviation (SD)

Run series	Char	SD	Liquid	SD	Gas	SD
SS, 50 g 600 °C	0.48	0.00	0.33	0.08	0.19	0.07
SS, 50 g 700 °C	0.45	0.00	0.26	0.04	0.29	0.04
SS, 20 g 700 °C	0.45	0.00	0.30	0.08	0.26	0.07
OF, 50 g 700 °C	0.53	0.02	0.14	0.02	0.33	0.03

Error bars represent experimental uncertainty in mass determination. The experimental uncertainty in char yield was low (0.5%) because the sample and char was confined to the sample basket and their mass was directly measured on the laboratory balance. Moreover, screening of the sample ensured that fine particles did not escape the basket.

Liquid and gas yield amounts had inherently greater uncertainty. For example, a loss or a gain of liquid during dismantling the apparatus or through condensation of water on the external surface of the condenser and seepage into collection flask, respectively, was observed. Furthermore, smaller absolute yields, with constant balance uncertainty, increased measurement uncertainty.

Gas sampling bags often contained a small amount of condensed tars. This indicated that some condensable pyrolysis products exited the apparatus as a vapour, but this amount was not quantified. The error bars in Fig. 5 do not include this error. Consequently, based on the conservation of mass, the calculated liquid fraction in the results is interpreted as the minimum liquid fraction while the calculated gas fraction is interpreted as a maximum value.

Standard deviation (SD) of char yields from SS was below 0.5%, which reflects the homogeneous composition of the feedstock. In contrast, the SD of OF samples was almost 4%. The SD values reflect the homogeneity of the former and the heterogeneity of the latter. This is despite the fact that the glass and metal fractions were removed from OF samples prior to pyrolysis. For SS feedstock, the particle size (pellets vs. ground material) was not observed (within SD) to influence mass yields.

### Proximate and ultimate analysis of feedstock and char

Feedstock had a modest higher heating value (dry basis) of 16.9 and 15.6 MJ kg^−1^ for SS and OF, respectively (Table 2). The difference in these values is a reflection of the higher volatile content of SS and its lower ash content. The ash content of OF is 42% compared to 32% for SS. The carbon content, which primarily determines the heating value of a fuel, is the same for both feedstock (Table 3).

**Table 2:** Proximate analysis of feedstock and pyrolysis char (dry basis). Fixed carbon content found by difference

Sample	HHV (MJ kg^−1^)	Volatiles (%)	Ash (%)	Fixed C (%)
SS	16.9	52.8	32.1	15.1
OF	15.6	42.2	41.5	16.4
SS char	11.7	3.2	69.4	27.4
OF char	12.2	3.9	64.2	31.9

**Table 3:** Ultimate analysis of feedstock and pyrolysis char (dry basis). Oxygen content found by difference

Sample	C (%)	H (%)	N (%)	S (%)	O (%)
SS	37.28	5.50	5.67	1.01	17.94
OF	37.48	4.13	2.48	1.44	16.12
SS char	28.68	0.09	1.94	0.41	− 0.41
OF char	34.96	0.42	1.13	2.41	− 3.14

The sewage sludge used in this study had typical characteristics. Typical sewage sludge, an average of 32 values reported in 18 studies, has a higher heating value of 16 MJ kg^−1^ (dry basis), a volatile matter content of 48.4%, a fixed carbon content of 7.6%, and is 44% ash (Syed-Hassan et al. 2017).

As waste material, both feedstock types and their chars have significant nitrogen (N) and sulphur (S) content. SS contain 5.7% N which is more than twice that of OF, being 2.5%. Less than half of the original nitrogen is preserved in the char after pyrolysis, 1.9 and 1.1% for SS and OF char, respectively.

OF char was observed to have 2.4% S, while the content in the feedstock was 1.4%. This result suggests that sulphur was conserved during pyrolysis and concentrated in the char, an observation consistent with other studies (Zhang et al. 2017). However, given the heterogeneous nature of the OF feedstock, the limited number of samples and the small sample size used in elemental analysis, this observation cannot be generalised. Moreover, the mass balance of elements from analysis did not sum up to 100%, and this is the reason for negative values for oxygen in the results.

### Pyrolysis gases

**Fig. 6:**
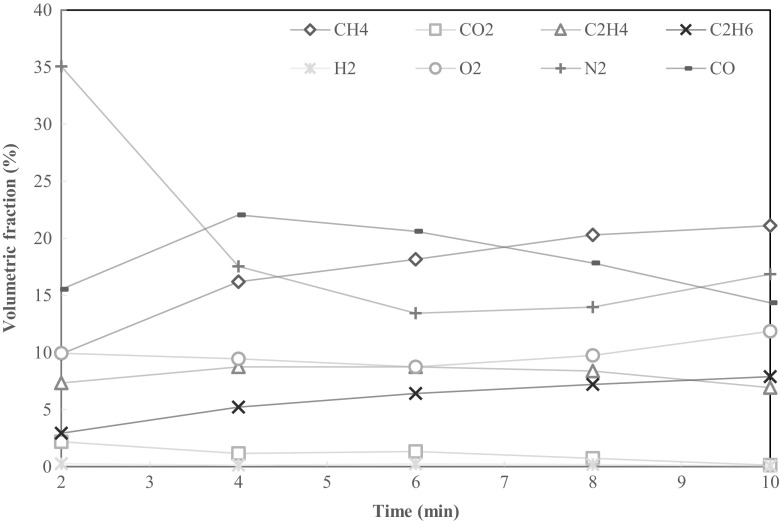
Volumetric fraction of pyrolysis gases from 50 g of SS at 600 °C

**Fig. 7:**
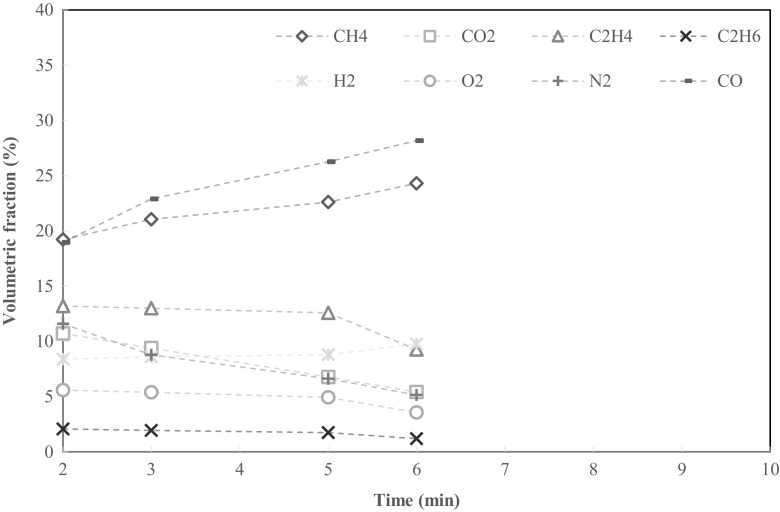
Volumetric fraction of pyrolysis gases from 50 g of OF at 700 °C

Results of gas analysis from SS and OF feedstock are presented in Figs. 6 and 7, respectively. Five samples, one every 2 min, were taken during SS pyrolysis. The combustible gas fractions, whose combined volume fraction range from 36 to 54% of observed gases, were predominantly carbon monoxide (CO), methane (CH_4_), ethylene (C_2_H_4_) and ethane (C_2_H_6_). The calculated LHV of the gas (Eq. 6) ranged from 11.8 to 19.1 MJ m^−3^. The fraction of carbon dioxide (CO_2_) declined from an initial 2%, and CO ranged from 14 to 22%, which indicates that carbon oxidation of the sample was insignificant.

Four gas samples were collected from OF pyrolysis, at 2, 3, 5 and 6 min. Compared to SS runs, the volume flow of gases from the reactor was significantly lower after midway through the run. This is explained by the higher pyrolysis temperature used and by the 10% lower volatile content of OF compared to SS. Gas sample collection from OF pyrolysis after the 6-min mark was not possible due to low flow rate. The combustible gas fractions ranged from 62 to 72% of observed gases and were CO, CH_4_, C_2_H_4_ and hydrogen (H_2_). The calculated LHV ranged from 18.2 to 21.0 MJ m^−3^ which is a significantly higher range than for SS pyrolysis—another reflection of the higher pyrolysis temperature. The fraction of carbon dioxide was some 6 to 11%, significantly higher than in SS pyrolysis, whereas the oxygen and nitrogen fraction were markedly lower.

Observations on pyrolysis gases were limited to relative volumes. The absolute amounts of each gas species produced from the feedstock cannot be determined without information on gas flow rates from the reactor during the pyrolysis run. Nonetheless, flow from the reactor was observed to be greatest during the first few minutes of pyrolysis.

## Conclusions

Physical characterisation of a pyrolysis reactor gives information on how process parameters influence heat transfer. This is essential knowledge in interpreting experimental results and in identifying differences between laboratory and pilot-scale processes and reactor design. Heat transfer conditions can be described accurately using the observable feedstock heating rate, the heat transfer coefficient and the *Biot* number.

The product distribution from pyrolysis of wastewater sludge and organic fines from municipal solid waste has been determined experimentally according to the principle of mass conservation. Pyrolysis char and gas yields were characterised and show potential for use as fuels in energy recovery from these two abundant feedstocks.

## Electronic supplementary material

ESM 1 (DOCX 143 kb)
