# Supplementary material for: Pyrolysis of wastewater sludge and composted organic fines from municipal solid waste: laboratory reactor characterisation and product distribution
Source: Environ Sci Pollut Res Int. 2018 Feb 26;25(36):35874–82. doi: 10.1007/s11356-018-1463-y (PMC6290694; doi:10.1007/s11356-018-1463-y)
Supplement: Supplementary file 1 — (DOCX 143 kb) [file 11356_2018_1463_MOESM1_ESM.docx]

**
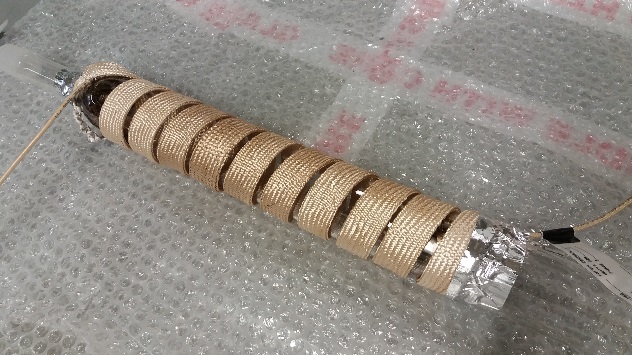
**

**Supplementary Figure**. The quartz tube reactor with heating tape wrapped around its exterior (before the application of insulation).
